# Climate change affects the distribution of diversity across marine food webs

**DOI:** 10.1111/gcb.16881

**Published:** 2023-10-10

**Authors:** Murray S. A. Thompson, Elena Couce, Michaela Schratzberger, Christopher P. Lynam

**Affiliations:** ^1^ Centre for Environment, Fisheries and Aquaculture Science (Cefas) Lowestoft Laboratory Lowestoft UK

**Keywords:** biodiversity, climate change scenarios, ecosystem structure and function, fish feeding guilds, habitat suitability, species distribution modelling

## Abstract

Many studies predict shifts in species distributions and community size composition in response to climate change, yet few have demonstrated how these changes will be distributed across marine food webs. We use Bayesian Additive Regression Trees to model how climate change will affect the habitat suitability of marine fish species across a range of body sizes and belonging to different feeding guilds, each with different habitat and feeding requirements in the northeast Atlantic shelf seas. Contrasting effects of climate change are predicted for feeding guilds, with spatially extensive decreases in the species richness of consumers lower in the food web (planktivores) but increases for those higher up (piscivores). Changing spatial patterns in predator–prey mass ratios and fish species size composition are also predicted for feeding guilds and across the fish assemblage. In combination, these changes could influence nutrient uptake and transformation, transfer efficiency and food web stability, and thus profoundly alter ecosystem structure and functioning.

## Graphical abstract

We model how climate change will affect the habitat suitability of marine fish species across a range of body sizes and belonging to different feeding guilds, each with different habitat and feeding requirements in the northeast Atlantic. Contrasting effects of climate change are predicted, with spatially extensive decreases in the species richness of consumers lower in the food web (planktivores) but increases for those higher up (piscivores). We therefore expect profound and complex changes in ecosystem structure and functioning, as shifts in the distributions of species performing critical ecosystem functions fundamentally alter how and where energy fluxes through the system.

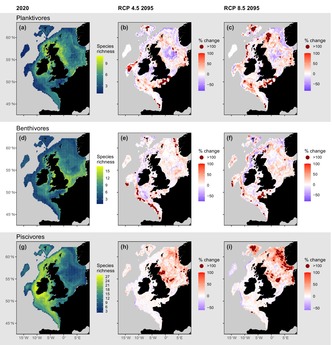

## 1 INTRODUCTION

Climate change is altering the distributions of many marine fish species, with widespread effects on biodiversity patterns (Hiddink & ter Hofstede, 2008; Magurran et al., 2015) and on ecosystem functioning (du Pontavice et al., 2020). Effects are predicted to increase (Fernandes et al., 2020; Jones & Cheung, 2015), and potentially jeopardise world food security (Pecl et al., 2017). Based on theory, modelling and empirical observations, climate change will favour smaller fish (Cheung et al., 2013; Daufresne et al., 2009). The incorporation of body size, a so called ‘super trait’ useful to assess change in food web structure and energy flux (Brose et al., 2019; Petchey et al., 2008), has improved model predictions of marine fish species distributions (Fernandes et al., 2013, 2020). Moreover, categorising fish using their species identity and body size into so called ‘feeding guilds’ associated with different prey has provided empirical evidence of changes in ecosystem structure and functioning in response to a range of environmental and anthropogenic drivers (Garrison & Link, 2000b; Thompson et al., 2020). Yet, predictions for how changes in species composition and size structure will affect the diversity of feeding guilds in response to climate change have not been made. This is despite feeding guilds being widely advocated in support of environmental status assessment (ICES, 2018; Rombouts et al., 2013).

Fish are key intermediate consumers in marine ecosystems because they link basal resources to top predators and occupy the majority of predatory roles above zooplankton, including the role of apex predator in many cases (Engelhard et al., 2014; Lynam et al., 2017). Fish also typically have different habitat and feeding requirements and thus occupy different roles in an ecosystem as they grow (Katara et al., 2021), starting in the planktivore feeding guild lower in the food web, with some developing into intermediate (benthivores) and higher predators (piscivores) as they go through ontogeny (Garrison & Link, 2000a; Thompson et al., 2020). Systematic differences in the response of typically smaller‐bodied fish, feeding lower in the food web (planktivores) relative to those bigger, which utilise different resources (benthivores) and feed higher up the food web (piscivores), could profoundly alter ecosystem functioning. This is because changes in diversity and body sizes across the food web can influence nutrient uptake and the efficiency of communities in converting nutritional resources into biomass (Cardinale et al., 2012; Wang & Brose, 2018).

Global‐scale studies which predict change in fish distributions in response to climate change typically focus on exploited species because of the wealth of distributional and biological data available for them via catch reports (du Pontavice et al., 2020; Jones & Cheung, 2015). Catch data, however, provide a skewed picture of biodiversity change because fishers target commercial species and sizes, under‐sample or underreport others, and are not obliged to follow consistent survey methods (Viana et al., 2013; Zhou et al., 2010). Internationally coordinated fisheries‐independent surveys offer a complementary perspective and provide a more comprehensive, albeit smaller‐scale, picture of biodiversity change (Magurran et al., 2015; global fish survey data with body size information are not yet readily available). Such surveys can be complemented with highly resolved size, life history and stomach content data (Thompson et al., 2020). Together, such data provide empirical observations to underpin predictions of fish species and sizes useful to understand changes in food web structure and ecosystem functioning. They have the added advantage of being collected specifically to inform regional ecosystem‐based management plans within which conservation efforts are coordinated.

The scaling relationship between predator and prey mass, often measured using predator–prey mass ratios (henceforth PPMRs), constrains energy flow through ecosystems (Barnes et al., 2010; Schneider et al., 2012). Predators which have many weak interactions by feeding on relatively small prey with high PPMRs can help to maintain stability in food webs (Otto et al., 2007; Rooney et al., 2006) and ecosystem functioning (Schneider et al., 2012; Wang & Brose, 2018) because they dampen strong oscillatory dynamics. As such, high PPMR interactions tend to mitigate perturbations from climate change, among other stressors (Binzer et al., 2016). PPMR estimates show significant interspecies variation due to differing foraging strategies, such that change in the composition of fish species can be the biggest driver of change in community‐wide PPMR (Reum et al., 2019). The ability to detect systematic change in fish with relatively high PPMRs in response to climate change would therefore be useful to better understand how changes in the fish assemblage may affect ecosystem functioning. Yet, predictions for how climate change could affect community‐wide PPMR via altered species and size composition have not been possible at large scales before because of insufficient data. Recent published inventories of stomach content information documenting predator and prey size and taxonomic information now contain the relevant data to make such predictions possible (e.g. Pinnegar, 2019).

We focus on the northeast Atlantic shelf seas where there are spatially and temporally extensive fisheries‐independent surveys (Lynam & Ribeiro, 2022), detailed feeding guild allocations (Thompson et al., 2020) and life history data (Thorson et al., 2017), complemented by model projections of changes in marine physics, biogeochemistry and the lower trophic levels of the marine food web (https://cds.climate.copernicus.eu/). We use Bayesian Additive Regression Trees (BART) to model the environmental requirements of species size classes which are grouped into feeding guilds and then test the following hypotheses: (i) climate change affects habitat suitability asynchronously for feeding guilds (i.e. change in species richness is unevenly distributed across the food web); (ii) changes in species and size composition are associated with widespread decreases in the mean maximum length indicator for fish and (iii) widespread decreases in PPMRs. Testing these hypotheses allows us to identify those ecosystem components and geographical areas most vulnerable to climate change.

## 2 METHODS

### 2.1 Survey data

Observations of fish species and sizes published on ICES DATRAS (https://www.ices.dk/data/data‐portals/Pages/DATRAS.aspx) were obtained for surveys of the Northeast Atlantic for the period 1983–2020. These data have been processed into a data product by Lynam and Ribeiro (2022) that is available publicly. Specifically, we make use of otter trawl data from the Greater North Sea (OSPAR Region II, https://www.ospar.org/convention/the‐north‐east‐atlantic) collected by international parties (i.e. International Bottom Trawl Survey, quarters 1 and 3) and by France (quarter 4); and from the OSPAR Celtic Seas region (III), that encompasses the western side of the British Isles collected by Northern Ireland (quarter 1) and by France, Northern Ireland and Scotland (quarter 4). We also make use of beam trawl data for specific demersal taxa (*Ammodytes*, *Limanda limanda*, *Microstomus kitt*, *Pleuronectes platessa*, *Scophthalmus maximus* and *Solea solea*) from the Greater North Sea collected by the Netherlands, Germany and England (quarter 3) and Celtic Seas region collected by England (Western Channel, quarter 1, and Bristol Channel and Irish Sea, quarter 3). Observations for those taxa were removed from the otter trawl data so as not to confound predictions with differences in gear‐specific observation biases. There were 44,103 unique hauls spanning years 1983–2020 with corresponding environmental data (see Table S1 for information on specific surveys and sample distributions).

### 2.2 Feeding guilds

Feeding guilds were allocated based on classifications following Thompson et al. (2020). In brief, a collation of trophic interactions spanning the northeast Atlantic shelf seas has been applied to define feeding guilds by grouping fish species size classes (we use the taxonomic level of Gobiidae and Ammodytes for taxa that are not consistently identified to species) that have prey taxa in common, and whose prey differentiate them from other predator guilds based on cluster analysis. Taxon‐specific size categories were defined as: <3 cm as larvae; small juvenile fish between 3 cm and half of length at maturity; juvenile‐medium fish from half of length at maturity to length at maturity; medium fish from length at maturity to half‐maximum length; and all remaining larger fish as large. Taxon‐specific length at maturity and maximum length (i.e. asymptotic length at infinity) were estimated using the R package *Fishlife* (Thorson et al., 2017). The stage of maturity, which has been shown to change in response to fishing and predation (Forestier et al., 2020) is not used here directly to inform our predictions; rather, each species was grouped into the above five size classes. Each species could thus shift guild through ontogeny, as many do (Thompson et al., 2020), but our results are not contingent on exactly when a species is mature.

PPMRs were based on directly observed predator and prey masses in stomach content data, where available (ICES, 1997; Pinnegar, 2019). Where predator mass was missing, this was estimated using published length–mass relationships (Silva et al., 2013). Where fish prey mass was missing, we used typical fish prey length to estimate prey mass based on length–mass relationships (after Pinnegar, 2019). The typical prey length of fish is estimated as:

$$\text{Prey length}=\left(0.2057\times \text{predator length}\right)+1.618.$$

For prey other than fish, we used mean size information from survey data for prey taxa where available (i.e. contained in Pinnegar, 2019, and references therein). PPMR was estimated for each stomach sample by taking a mean of individual PPMR values (predator mass/individual prey mass), weighted by prey biomass, which is appropriate to assess energy flux (Reum et al., 2019). PPMR was then calculated for species within guilds by taking the mean of these biomass‐weighted PPMR values across unique predator samples (Table S2). Because we are not modelling change in all prey species and sizes (e.g. zooplankton), predicted changes in PPMR reveal whether fish species and sizes with systematically different PPMRs have contrasting responses to climate change, as opposed to a prediction of community PPMR itself.

**FIGURE 1:**
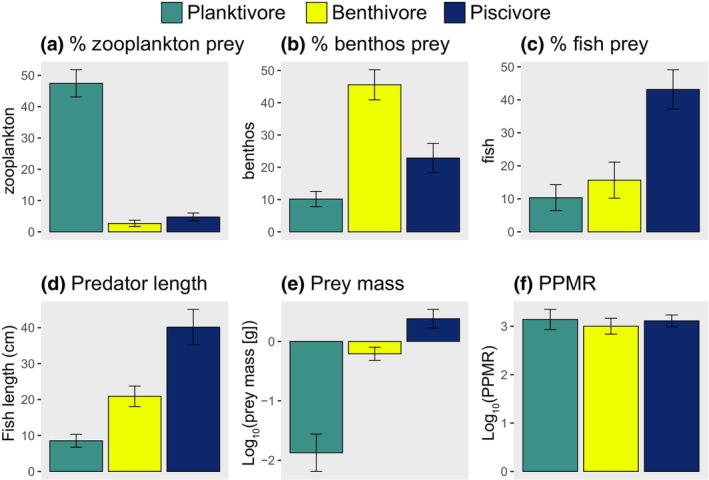
Differences between feeding guilds in: % biomass contribution of zooplankton (a); benthos (b); and fish prey (c; see Figure S1 for remaining prey biomasses); predator length (d); individual prey mass (e); and biomass‐weighted predator–prey mass ratio (f). Values are based on feeding guild‐level means taken across species (Table S2), error bars represent standard error.

We use a relatively simple set of feeding guilds where we take a higher split in the classification tree than those more complex guilds used by Thompson et al. (2020), and these can be described as planktivores, benthivores and piscivores because of the mean relative % biomass contributions of those prey groups to their diets (Figure 1, Figure S1; Table S2). Prey were assigned to functional groups in R version 4.02 (R Core Team, 2020) after Webb and Vanhoorne (2020) using the ‘worrms’ package (Chamberlain, 2019). Planktivores are typically smaller‐bodied fish that feed on relatively small prey lower in the food web, whereas benthivores are intermediate in size, feed on intermediate sized prey and piscivores are the largest and feed on relatively large prey. All guilds have similar mean PPMRs. We used these simplified guilds so that we could elegantly capture a broad set of ecosystem components while also explore a complementary suite of responses and two different climate scenarios. Feeding guild classifications were applicable to 92.5% of the biomass observed in the otter trawl survey data which included 78 species (not including those targeted using beam trawls). However, many rare species observed in the surveys, representing 7.5% of the biomass surveyed using otter trawls, remained unclassified due to limited stomach content information. Our perspective of fish biodiversity was therefore weighted towards predators contributing most to community biomass and ecosystem functioning.

### 2.3 Climate projections and environmental change gradients

Environmental projections from 2006 to 2100 were derived from the coupled marine ecosystem models POLCOMS/NEMO‐ERSEM. ERSEM v15.06 (European Regional Seas Ecosystem Model; Butenschön et al., 2016). This is a complex lower trophic‐level marine ecosystem model that simulates bacteria, four phytoplankton and three zooplankton functional groups, and includes a fully resolved diurnal cycle, variable carbon to chlorophyll ratios and independent nutrient pools for carbon, nitrogen, phosphorous and silicate. ERSEM was coupled to the ocean circulation model POLCOMS (Proudman Oceanographic Laboratory Coastal Ocean Modelling System; Holt & James, 2001; Holt et al., 2001) running at a resolution of 0.1°, and together providing projections of changes in marine physics, biogeochemistry and the lower trophic levels of the marine food web for our study area (Kay, 2020). These datasets are publicly available to download from the online catalogue of the Copernicus Marine Environment Monitoring Service at https://cds.climate.copernicus.eu/cdsapp#!/dataset/sis‐marine‐properties.

We focus on two emission scenarios (representative concentration pathways, RCPs) developed for the Intergovernmental Panel on Climate Change (IPCC)'s fifth phase of the Coupled Model Intercomparison Project (CMIP5): RCP 4.5, the ‘stabilization scenario’, characterised by medium emissions and high mitigations, and the ‘no mitigation policy’ scenario RCP 8.5, derived from high fossil fuel emission and low mitigations. Together they provide information on the implications of stabilising emissions versus inaction on climate change. Global Earth System models are also available, but these require statistical downscaling using regional models so that they capture specific regional processes (Holt et al., 2014). Here we use a specifically developed model for the region that provides the range of environmental data required for the study.

We use projections of temperature, salinity, pH, nitrate, phosphate, dissolved oxygen, current velocity, chlorophyll, gross primary production, non‐living organic carbon, zooplankton carbon concentration and secondary carbon production by zooplankton in our predictive habitat models. For temperature, salinity, current velocity, dissolved oxygen and pH both surface and bottom mean annual averages were considered, and in the case of temperature, also the difference between bottom and surface values to account for stratification. For chlorophyll, gross primary production, non‐living organic carbon, phosphate, nitrate, zooplankton carbon concentration and secondary carbon production by zooplankton the total across the water column was used, rather than surface or bottom values. We also include depth (from the General Bathymetric Chart of the Oceans GEBCO; www.gebco.net, at 15 s resolution), distance to coast and substrate composition (median grain size and percentages of mud, sand and gravel from Wilson et al., 2018) to capture key spatial gradients that affect habitat suitability for fish. Data on fishing at sufficiently high spatial resolution are only available for recent periods starting circa 2009 which corresponds with only 45% of the survey data. Hence, we chose not to include fishing pressure information and include all survey data available to increase the power of our statistical approach with the caveat that impacts of fishing on the ranges of species within guilds were not accounted for. This also influenced our decision to model distributions (i.e. presence/absence data, see below) as opposed to abundance, since the impact of fishing is more pronounced in terms of abundance, with many species whose populations have been depleted by fishing still regularly observed in surveys, albeit in lower numbers (e.g. North Sea cod; Horwood et al., 2006).

All environmental data were processed onto a 10 × 10 km^2^ grid, and because an annual mean of, for example, temperature, does not capture the environmental variability that ultimately determines the thresholds within which biota must survive; for temperature, salinity, pH, oxygen and current speed we also include the standard deviation of the 12 monthly means in each year, for all locations within a radius of 75 km of each grid cell, in order to provide a measure of spatio‐temporal heterogeneity. For the environmental variables where surface and bottom values were extracted, we use sea surface values to model habitat suitability for planktivores, which are largely pelagic species, and seabed values for the benthivores and piscivores which are largely demersal species. Pairwise Pearson correlation coefficients were computed for the set of environmental variables used to model the habitat of planktivores and of non‐planktivores separately, to assess multicollinearity. Variables were removed if they correlated with another >0.7 (Figure S2, also showing the final set of variables used to train the models).

### 2.4 Habitat suitability modelling and estimating uncertainty

**FIGURE 2:**
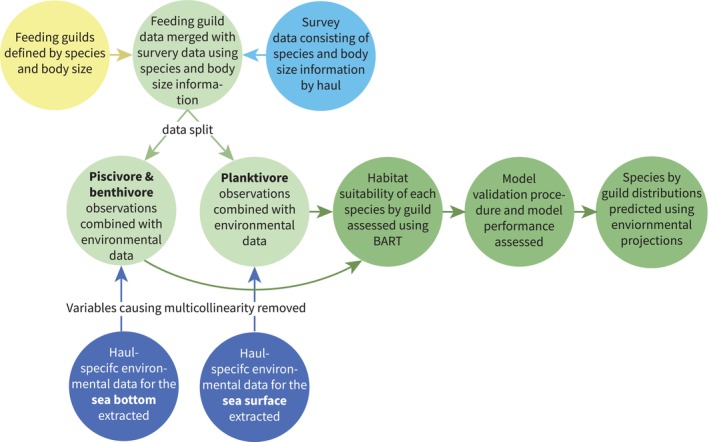
A step‐by‐step guide showing how different data were combined and analysed to predict species‐guild distributions.

The beam and otter trawl survey data were processed using the 10 × 10 km^2^ study grid. This grid resolution was used because the hindcast of the environmental data (CERES, 2018) and the forecast data from Copernicus have a resolution of approximately 11 km, whereas the bathymetry data have a higher resolution (<10 km). Our outputs were therefore limited by the climate projections made by general circulation models. Grid cells where species size classes classified into guilds (henceforth ‘species‐guild’; see Table S3) were observed on a particular year were classified as ‘presence’ sites and ‘absences’ were assumed where and when a survey using the same gear (beam or otter trawl) had failed to find the species‐guild (i.e. our models use and predict the presence or absence of species‐guilds as opposed to abundance or biomass). Models for the sandeels (*Ammodytes*) and flatfish *L. limanda*, M. kitt, *P. platessa*, *S. maximus* and *S. solea* were trained using presences and absences from surveys using beam trawls, while those for other species were trained solely with otter trawl survey data. Since data from different gears were not combined it was not necessary to account for differences in catchability. All gridded environmental data were extracted to match the grid cell and year that the survey data were collected, meaning each haul had unique environmental information (Figure 2 provides a visual schematic outlining the steps we took). The environmental requirements of each species‐guild are assessed using BART (Chipman et al., 2012). BART is a method based on an ensemble of classification tree models, each built by sequentially splitting the data into two groups based on the value of the explanatory variables. BART can account for nonlinear relationships and interactions between explanatory variables, much like all tree‐based techniques. However, it differs from other frequently used tree ensemble models, such as Random Forest or Boosted Trees, in that the sequence of trees is built relying on a Bayesian probability model, with the tree structure dictated by priors and a likelihood for the data in the terminal nodes of the trees. The sequential process of building BART trees means each new tree has an increasingly weaker effect on the final predictions. While the application of BART for species distribution modelling is relatively novel and not widely extended yet, it has been shown to have similar or better performance than the majority of more popular machine learning techniques, including Random Forest Analysis (Chipman et al., 2012). The Bayesian approach of the BART method also allows for an estimation of prediction error, which is lacking for most traditional species distribution modelling techniques. The models were trained in R using the ‘embarcadero’ package (Carlson, 2020).

We train models for all combinations of species‐guild for which at least 40 presences were available in the training dataset, resulting in 71 different models (see Table S3 for a full list of the models and the number of presences available for each). We assess model performance in both space and time. Spatial performance was analysed via eightfold block cross validation using the R library ‘blockCV’ (Valavi et al., 2019). In this approach, the study region is divided into a coarse grid, with the resolution of the grid taking into account the spatial autocorrelation of the environmental variables (we used data for the year 2000 to assess this). These spatial blocks were then assigned numbers from 1 to 8. For each species‐guild, eight additional models were trained, using data from blocks from all numbers except one, leaving a different number out each time. Each model is then used to predict on the blocks with the number that was excluded when training that particular model (called ‘validation’ data). Each model is therefore predicting to a ‘novel area’, outside the spatial boundaries where it was trained. Prediction data of all the eight models cover the entire study region and were pulled together to assess model performance. Temporal performance of the models was assessed by training a new model that excluded the last 5 years of survey data (2015–2019), using the resulting model to predict to those 5 years, a ‘novel time period’ for that model. It was additionally required that at least 10 presence records fell within the validation period. This was easily achieved by a majority of our models (e.g. 67 of the 71 models we trained had at least 400 presence records and the majority had many more, see Table S3), but for three of the models (*Anguilla anguilla*—Benthivore, *Belone belone*—Planktivore and *Scophthalmus rhombus*—Benthivore) the year for the split had to be lowered to 2007, 2013 and 2006 respectively in order to achieve 10 presence records.

For both the spatial and temporal validations (Valavi et al., 2019) we computed three measures of model performance: the area under the curve (AUC) of the receiver operating characteristic (ROC) plot, the AUC of the Precision–Recall (PR) plot (He & Garcia, 2009) and the Miller slope (Miller et al., 1991; Table S3). We required that models achieved AUC scores of the ROC curves of 0.65, for both the temporal and spatial validation. All of the 71 trained models met this requirement. Our results therefore represent change in the composition of feeding guilds based on species and sizes that are observed in the trawl surveys and whose distribution can be modelled with a sufficient level of confidence. Table S3 lists species and sizes by feeding guild with their respective model performance scores and all modelled projections can be found in Couce and Thompson (2023). For each model, a presence/absence threshold was chosen as the value that maximised each model's sensitivity plus specificity in the test data of the spatial eightfold block cross validation. We chose to model species‐guilds, as opposed to species size classes, because feeding guilds are a data‐driven approach to determining key shifts in species life history. Variable importance for each BART model was assessed as the proportion of times each variable was used in the split of the branches across all individual tree models, and was then averaged for all models in each feeding guild to reveal which predictors best explained the distributions of species within guilds.

Future projections were based on the predicted presence of species‐guilds at each grid cell under specific RCPs and years. Species richness was estimated by summing species within feeding guilds to assess specific components of the food web or across guilds for a community‐wide perspective. Species‐level maximum length estimates made by Thorson et al. (2017) were used to assess change in fish size composition by taking the mean maximum length across all species present. PPMR estimates were determined by taking a mean across species within each feeding guild and across feeding guilds for a guild‐level and community‐wide perspective of change, respectively.

We then test our hypotheses in two ways to assess large‐scale and fine‐scale temporal change respectively. First, using Kruskal–Wallis tests between cell‐level values in 2020 compared with those in 2095 to assess whether change in each response, for example, planktivore species richness, was significant across the study area. Second, we asses finer scale temporal correlations using Kendall's τ trend analysis based on the relationship between the various responses and year across each grid cell (*n* = 13,318 cells over 5‐year intervals from 2020 to 2095). Kendall's τ scores are presented for each grid cell to show regions of temporal change and thus explore contrasting directions of change which may go undetected at the scale of the study area. Kendall's τ scores of −1 to +1 represent a 100% probability of a decreasing or increasing trend respectively. Both statistical approaches we use to test our hypotheses are rank based and nonparametric because our response variables were not normally distributed and could not be readily transformed to meet parametric model assumptions. Using Kendall's τ has the added benefit of detecting correlations which may be nonlinear, since temporal change in the responses we measure could take any smoothly varying (curving) function. We focus these tests and much of our results on RCP 4.5 because our hypotheses are not associated with the differences caused by the different scenarios, but rather the contrasting effects of climate change across the food web. We then compare spatial patterns of change between RCP 4.5 and RCP 8.5 to draw attention to the level of change expected between different mitigation options.

## 3 RESULTS

### 3.1 Change in species richness

**FIGURE 3:**
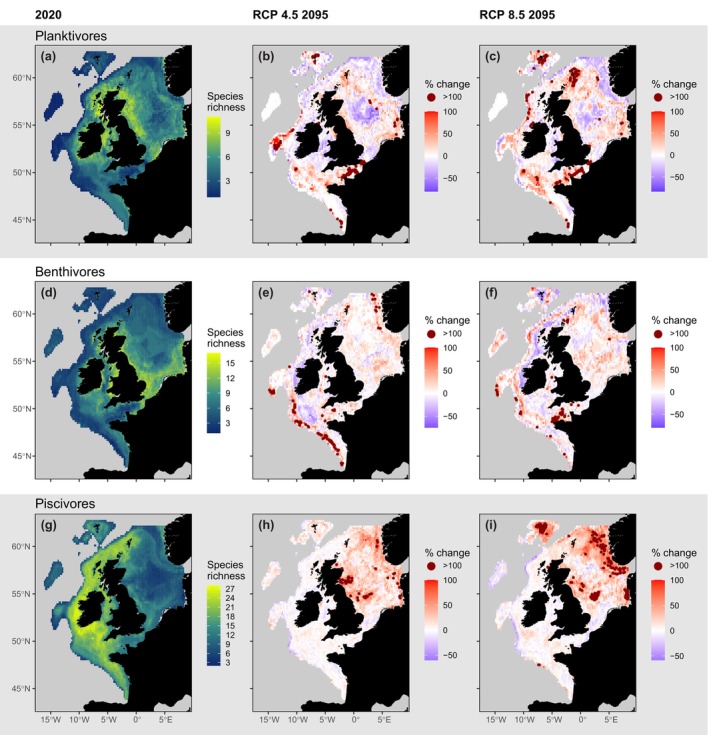
Feeding guild species richness in 2020 (a, d, g) and % change in richness between 2020 and 2095 based on RCP 4.5 (b, e, h) and RCP 8.5 (c, f, i) generated using BART species distribution models. Particularly high values of increase by >100% are highlighted in dark red.

**FIGURE 4:**
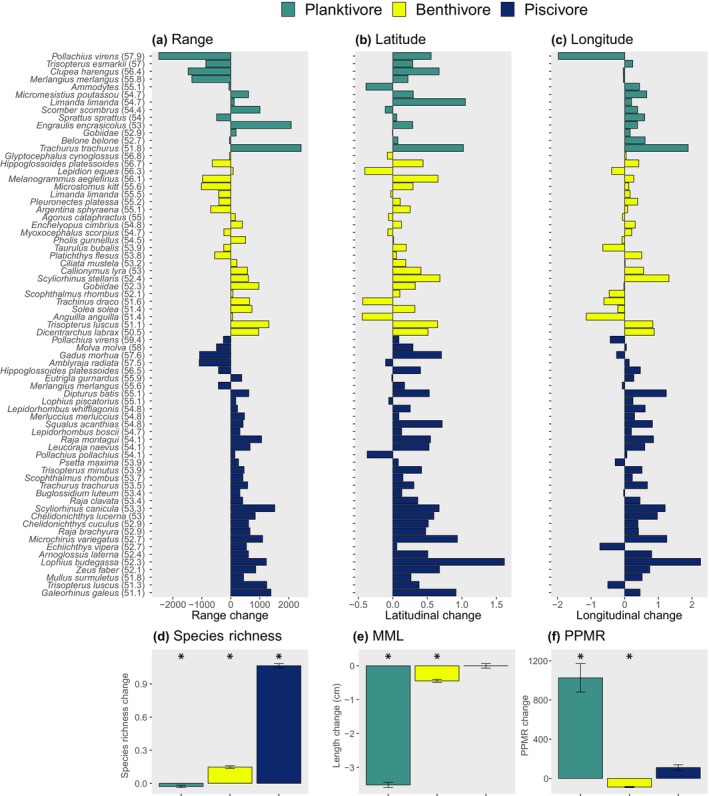
Predicted change in the distribution of species assigned to feeding guilds, feeding guild species richness, mean maximum length (MML) and predator‐prey mass ratios (PPMR) from 2020 to 2095 based on RCP 4.5. Top row: species are ordered along the y‐axis by feeding guild and then their mean latitudinal values (in parentheses). Change in range (a) represents change in the number of cells occupied across the study region, each cell corresponding to an area of 100 km^2^. Latitudinal (b) and longitudinal (c) change represent shifts in the mean latitudinal and longitudinal values of cells occupied by species respectively. Species which appear multiple times on the *y*‐axis switch guilds through ontogeny, such as juvenile planktivorous dab (*Limanda limanda*) which develop into benthivores at larger size classes and can have differing habitat requirements (note contrasting latitudinal changes). Bottom row: mean cell‐level change in feeding guild species richness (d), MML (e) and PPMR (f), with error bars showing standard error and * indicating significant change between 2020 and 2095 values based on Kruskal–Wallis tests.

**FIGURE 5:**
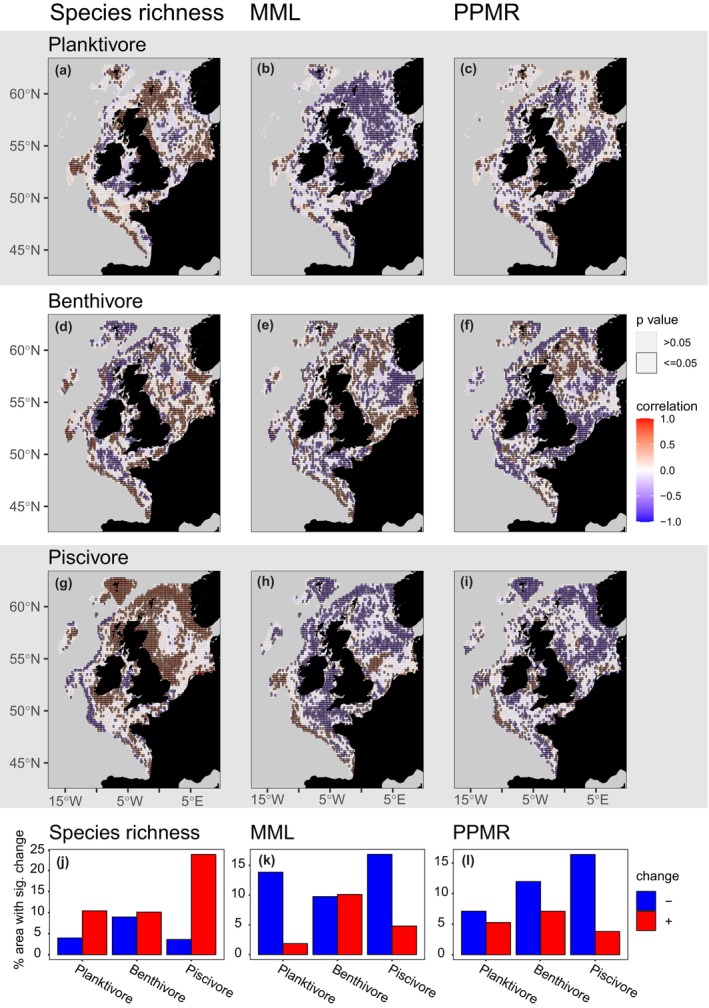
Temporal correlations in feeding guild species richness (a, d, g), mean maximum length (MML; b, e, h) and predator–prey mass ratio (PPMR; c, f, i) over 5‐year intervals from 2020 to 2095 under RCP 4.5. Temporal increases are shown by red cells (Kendall's τ correlation values between 0 and +1), declines by blue cells (correlation values between 0 and −1), and cells with significant correlations have a black border. The bottom row shows the % of cells with a significant increasing (red) or significant decreasing (blue) correlation in species richness (j), MML (k) and PPMR (l).

Substantial change in the spatial distribution of fish species richness in response to climate change is predicted, with contrasting directions of change across feeding guilds (Figures 3, 4, 5). At the large‐scale, planktivore species richness will decrease (*χ* ^2^ = 3.93; Df = 1; *p* = .047), increase for benthivores (*χ* ^2^ = 13.58; Df = 1; *p* = <.001) and increase for piscivores (*χ* ^2^ = 246.79; Df = 1; *p* = <.001) under RCP 4.5 by 2095 (Figure. 4d). At finer scales however, all feeding guilds show contrasting directions of change in species richness with more cells showing significant positive trends than significant negative trends (Figure. 5). In general, change is predicted to be driven by range contraction of more northerly distributed species and range expansion of those more southerly distributed, with more species expanding in range, moving north and east, than contracting (Figure 4a–c). Variable importance across our models shows that temperature, distance to coast, bathymetry (i.e. depth) and the % of different substrates were generally important predictors, while current speed, total non‐living carbon concentration and nutrients were often least important (Figure S3; see also Figure S2 for covarying predictors which were excluded). Decreases in planktivore richness were most notable in the central North Sea with up to 80% declines (−6 species; Figures 3 and 5) with increases of up to 300% (+5 species) also evident, particularly in southern and western areas of the study region, where species richness was previously low. In contrast, under those same projections, spatially extensive increases in piscivore richness were predicted, with the largest increases of up to 250% (+12 species) in the eastern and central North Sea, and some localised and more minor declines largely occurring in the west of the study region of up to 60% (−8 species). Benthivore richness showed large areas of change in each direction, with increases of up to 400% (+6 species) in deeper waters in the Atlantic and Norwegian trench, and in the English Channel, and decreases of up to 75% (−6 species) off the west coast of Ireland and more broadly across shelf‐sea areas of the Celtic Sea. Prediction errors revealed that uncertainty in our predictions was higher on average for planktivores relative to other guilds in 2020, and higher for planktivores and piscivores relative to benthivores in 2095 (Figure S4).

Predicted increases in species richness across feeding guilds (*χ* ^2^ = 195.09; df = 1; *p* < .001; Figure S5) were largely driven by change in the most species rich piscivore guild (Figures 3, 4, 5). Species richness increases were greatest in the northeast (up to +100%; +17 species), particularly in the deeper waters around Norway, and south‐central and western areas of the North Sea, with less notable decreases in species richness in the north‐central North Sea, the Irish Sea and off the west coast of Ireland (up to ‐36%; ‐9 species) by 2095 under RCP 4.5. When assessing community‐wide richness, increases in the piscivore guild largely obscured regionally contrasting patterns of change, including widespread losses of planktivore and benthivore species that are critical to maintaining ecosystem functioning.

Compared with RCP 4.5, the patterns of change in the richness within and across feeding guilds under RCP 8.5 were broadly similar by 2095, but the magnitude was greater (Figure 3 and Figure S5). Change in planktivore richness ranged from −83% (−5 species) to +400% (+7 species), in benthivore richness from −80% (−7 species) to +233 (+7 species), in piscivore richness from −58% (−8 species) to +300% (+13 species) and changes in overall species richness ranging from −53% (−10) to +106% (+17).

### 3.2 Change in MML and PPMR

Changes in the spatial distributions of species within guilds were predicted to affect the species composition of feeding guilds and community composition overall (i.e. as characterised by the MML indicator) and PPMR by 2095 under RCP 4.5 (Figures 4, 5 and Figure S5). Large‐scale decreases in MML were highest for planktivores (*χ* ^2^ = 736.20; Df = 1; *p* = <.001), followed by benthivores (*χ* ^2^ = 15.18; Df = 1; *p* = <.001), with no overall change for piscivores (*χ* ^2^ = 1.54; Df = 1; *p* = .215; Figure 4). At finer spatial scales, there were widespread declines in MML for planktivores and piscivores, with roughly equal areas of contrasting change for benthivores (Figure 5). Across feeding guilds, there was no overall direction of change in MML (*χ* ^2^ = 0.00; df = 1; *p* = .961), but large areas of change are predicted in each direction, with increases of up to 58% (+29 cm) in the southeast and western North Sea but decreases of up to 36% (−29 cm) most notable in the central‐eastern North Sea (Figure S5).

At the large‐scale, PPMR increased for planktivores (*χ* ^2^ = 13.51; Df = 1; *p* = <.001) but decreased for benthivores (*χ* ^2^ = 75.09; Df = 1; *p* = <.001) with no overall change for piscivores (*χ* ^2^ = 0.00; Df = 1; *p* = .985; Figure 4). At the finer spatial scale, PPMR decreased over more cells than it increased for all feeding guilds, revealing that species richness increases were typically driven by fish with relatively low PPMR (Figure 5). PPMR across feeding guilds changed in each direction showing no overall direction of change (*χ* ^2^ = 1.01; df = 1; *p* = .315; Figure S5), with decreases in the eastern North Sea of up to 90%, (−20,267) and increases in parts of the central North Sea of up to 1301% (+21,247). Under RCP 8.5, the magnitude and patterns of change across feeding guilds predicted by 2095 were largely similar compared to RCP 4.5 but with more widespread declines in MML and PPMR (Figure S5), with change in MML ranging from from −46% (−27 cm) to +71% (+24 cm) and change in PPMR from −91% (−19192) to +1040% (+19584).

## 4 DISCUSSION

While it is widely acknowledged that climate change is impacting marine fish species distributions and size compositions (Fernandes et al., 2013; Hiddink & ter Hofstede, 2008; Jones & Cheung, 2015), this study is the first to demonstrate that changes in the spatial patterns of species richness will be different across feeding guilds in response to climate change. Species within feeding guilds that are predicted to increase in range do not simply occupy space left by species within the same guild decreasing in range (Figures 3 and 4). Instead, those increasing in range are predicted to prosper in other areas of the ecosystem, with some regions experiencing contrasting directions of change in species richness across the food web (Figures 3 and 5). These changes will deplete species in some areas where they perform critical ecosystem functions (e.g. planktivorous herring, *Clupea harengus* and Norway pout, *Trisopterus esmarkii*), while bolstering others (e.g. planktivorous anchovy, *Engraulis encrasicholus* and Atlantic horse mackerel, *Trachurus trachurus*; Figure 4, Figure S6), and thus fundamentally alter how and where energy fluxes through the system.

Trophic transfer efficiency (du Pontavice et al., 2020) and the number of feeding links, especially for predators (i.e. predator generality; Albouy et al., 2014) have been predicted to decrease in marine ecosystems under climate change. Evidence from meta‐analyses also shows that biodiversity loss impairs both nutrient uptake and the efficiency of communities in converting nutritional resources into biomass (Cardinale et al., 2012; Worm et al., 2006). Contrasting directions of change in responses across the food web could therefore have profound effects on ecosystem structure and functioning. For the first time, our study provides evidence for decreasing species richness and change in size structure in a key energy pathway, planktivory by fish, over large areas which could undermine energy transfer between basal resources and top predators. Conversely, the efficiency of energy uptake and biomass production could be elevated for piscivores where richness and, in some cases, ultimate body size, is anticipated to increase (Figure 5; Wang & Brose, 2018). Planktivores with high PPMR (which typically feed on relatively small prey, e.g., herring; Table S2) are predicted to increase in some areas, but across much of the study region and all feeding guilds PPMR is predicted to decrease (predators typically feeding on relatively larger prey; Figure 5). Similarly, the composition of fish will largely shift towards those with decreased ultimate body size. Spatially extensive reductions in PPMR and body size across the food web could strengthen oscillatory dynamics between interacting fish species and thereby undermine food web stability (Otto et al., 2007; Rooney et al., 2006) and ecosystem functioning (Schneider et al., 2012; Wang & Brose, 2018). Future research could explore the consequences of these contrasting changes in species richness, PPMR and MML across the food web, for example, via dynamical spatio‐temporal food web modelling, to help understand how food web dynamics might affect biotic responses to these environmental change drivers.

Community‐wide increases in species richness (Figure S5; Hiddink & ter Hofstede, 2008) will be largely driven by piscivores (Figures 3, 4, 5), but this obscures widespread declines in more northerly distributed planktivorous fish with relatively large ultimate body size (i.e. compared to those planktivore species expanding in range) that play a critical role in regulating ecosystem dynamics and functioning across the northeast Atlantic shelf seas (Figures 3, 4, 5; Engelhard et al., 2014; Lynam et al., 2017). This is partly caused by diminishing suitable habitat for juvenile planktivorous gadoids that develop into piscivores as they mature (*Merlangius merlangus and Pollachius virens*), but also sandeels, sprat, herring and Norway pout, for instance, which remain planktivorous through ontogeny (Figure 4; Figures S6 and S7, the latter showing maps of temporal change in planktivorous fish MML where only species with adult planktivorous life stages are considered). Given planktivores tend to be smaller (Figure 1), and warming favours smaller fish (Daufresne et al., 2009), this finding is counterintuitive, and suggests that foraging strategies, as well as body size and habitat usage, play a central role in determining diversity patterns and organismal response to climate change (Siqueira et al., 2021).

There was no uniform response to climate change, with contrasting directions of change within and across feeding guilds. For instance, contrasting regional directions of change in benthivore species richness were equally widespread (Figure 5j). There were also apparent contradictions in the direction of change across spatial scales. For instance, increases in planktivore species richness were more spatially extensive than decreases (Figure 5j), but planktivore species richness decreased overall (Figure 4d). Similarly, decreases in planktivore PPMR were more spatially extensive than increases (Figure 5l), but increased overall (Figure 4f). These are the result of differences in the quantity (level of change) and the direction of change in space, that is, relatively large change in a small area with relatively minor change in the opposite direction over larger areas. Assessment across spatial scales was key to detect these nuances which are important to understand because different management may be needed in areas with contrasting directions and magnitudes of change.

Rather than using fisheries catch data, we use fisheries‐independent survey data to reveal changes in the distribution of specific life stages of a broad range of species (i.e. including but not exclusively commercially fished species) which feed at different levels in the food web. Our findings can help identify (i) species whose habitat conditions are changing at different rates between juvenile and adult life stages (e.g., dab, *Limanda limanda*; Figure 4a‐c) and (ii) regions where species richness and ecosystem functioning are predicted to change most in response to climate change and where environmental protection may be most warranted and most effective. This may include, for example, areas in the northern North Sea which are predicted to be a thermal refuge for many species in the future (Figure 3 and Figure S5), or more stable areas such as Atlantic facing coastal waters of the United Kingdom and Ireland where spatial protections could cover areas of high biodiversity into the future.

The species richness of planktivores was more sensitive to change in individual species because they had low overall species richness compared to the other feeding guilds. Community PPMR was also sensitive to change in a few planktivorous species with particularly high PPMR (Table S2). Relative to the other guilds, this highlights: (i) that planktivores, which represent the most prominent intermediate consumers (both numerically and in terms of biomass) that support higher predator populations in the region (Engelhard et al., 2014; Lynam et al., 2017; Wilson & Hammond, 2019), tend to be more vulnerable to climate change relative to other feeding guilds and; (ii) the directions of change in planktivore species richness and PPMR are less certain, given these are contingent on changes in relatively few species with generally higher uncertainty in their modelled distributions (Figure S4). Some small planktivorous species (e.g. pilchard, *Sardina pilchardus*) are poorly sampled by the scientific trawl surveys we make use of, as are the small planktivorous juveniles of species that later grow into benthivores or piscivores (e.g. pollack, *Pollachius pollachius*; Table S3; Thompson et al., 2020). Our results therefore represent changes in those species or life stages that are well‐sampled in the surveys, and whose distribution can be modelled with a sufficient level of confidence. In future, internationally coordinated stomach content sampling and survey effort directed towards small species and juvenile life stages will be needed to improve our understanding of the habitat requirements of fish critical to maintaining ecosystem functioning.

We predict substantial change across the fish component of the food web within each RCP scenario with greater magnitudes of change in species richness and more widespread declines in MML and PPMR under RCP 8.5 relative to RCP 4.5 (Figure 3 and Figure S5). Future work could consider more stringent climate mitigation scenarios, for example, RCP 2.6 for which projections from Copernicus do not currently exist, and multi‐model ensemble outputs as they become available. Categorising fish contained in global catch datasets into feeding guilds using stomach content datasets from marine ecosystems not considered here could also be attempted to test how changes across the food web will affect fisheries worldwide. Recent rapid advances in joint species distribution modelling could be applied to study how interactions between fish affect the distribution of diversity across the food web (Tikhonov et al., 2020), with careful consideration for how including size with species information could affect results. Furthermore, modelling guild‐level biomass, as opposed to the presence or absence of species and size classes alone, could pave the way for estimates of biomass distribution and energy flux that were not possible here and still represent a major challenge when predicting ecosystem change at finer spatial scales (Fernandes et al., 2020). Another significant future challenge, particularly relevant when predicting fish abundance and biomass, is the incorporation of fishing pressure which could be done via reconstructing effort where data are lacking (Couce et al., 2020) or using fisheries catches (Watson, 2017), for example, which cover the survey time series but are recorded at a coarse spatial scale.

Assessing change in functionally distinct feeding guilds has been widely advocated to support environmental status assessment (ICES, 2018; Rombouts et al., 2013), but not yet applied to predict change in the distribution of diversity across the food web in response to climate change. Using a suite of complementary responses across species within feeding guilds, we predict that there will be clear regions of change in species richness across the food web and community‐wide measures of species composition, size structure and predator–prey interactions. Such insights represent valuable information to help anticipate and mitigate climate change effects on marine ecosystems.

## 5 CONCLUSION

By modelling climate change effects on the habitat suitability of fish with different diets, we reveal that species richness will change at different rates and even in different directions across the food web over large areas. We therefore expect profound and complex changes in ecosystem structure and functioning, as shifts in the distributions of species performing critical ecosystem functions fundamentally alter how and where energy fluxes through the system. Importantly, our results show that regional increases in community‐wide species richness can mask the loss of species life stages that are critical to maintaining ecosystem functioning. These changes add to the evidence base that world food security could be jeopardised by climate change.

## AUTHOR CONTRIBUTIONS

Murray S. A. Thompson and Elena Couce conceived the ideas and designed the methodology; Murray S. A. Thompson, Elena Couce and Christopher P. Lynam collated and processed the data; Murray S. A. Thompson, Elena Couce, Michaela Schratzberger and Christopher P. Lynam analysed the data; and Murray S. A. Thompson led the writing of the manuscript. All authors contributed critically to the drafts and gave final approval for publication.

## FUNDING INFORMATION

MT, EC, MS and CL were funded by Cefas Seedcorn projects ‘Forecasting and valuing changes in food web structure and function in response to environmental change’ (DP427) and ‘Next generation Cefas biodiversity science: from individuals to ecosystems’ (DP433); MT and EC were supported via the Natural Environment Research Council grant NE/V017039/1; MT, EC and CL were supported via the European Union's Horizon 2020 research and innovation programme under grant agreement no. 869300 ‘FutureMARES’.

## CONFLICT OF INTEREST STATEMENT

The authors declare no conflict of interest.

## Supporting information


Data S1.

## Data Availability

The data we make use of are all publicly available, cited and displayed alongside URLs, where applicable. Our modelled projections of habitat for fish species within feeding guilds around North‐western Europe under climate change, from 2010 to 2095, can be found here: https://doi.org/10.14466/CefasDataHub.139.
