# Supplementary material for: Climate change affects the distribution of diversity across marine food webs
Source: Glob Chang Biol. 2023 Oct 10;29(23):6606–19. doi: 10.1111/gcb.16881 (PMC10946503; doi:10.1111/gcb.16881)
Supplement: Supplementary file 1 — Data S1. [file GCB-29-6606-s001.docx]

**SUPPORTING MATERIAL**

**Title:** Climate change affects the distribution of diversity across marine food webs

**Running title**: Climate change affects marine food webs

**Authors**: Murray S. A. Thompson^1^, Elena Couce^1^, Michaela Schratzberger^1^ and Christopher P. Lynam^1^

^1^ Centre for Environment, Fisheries and Aquaculture Science (Cefas), Lowestoft Laboratory, Pakefield Road, Lowestoft, Suffolk, NR33 0HT, UK

**Correspondence**: [murray.thompson@cefas.co.uk](mailto:murray.thompson@cefas.co.uk)

Table S1. Survey acronyms, countries responsible, gear used, spatial, temporal ranges (including quarter; Q), observed number of species used for this study from specific gear (spp), and number of hauls (*n*). See Lynam & Ribeiro^1^ for full description of surveys and data processing.

| **Acronym** | **Country** | **Gear** | **Area** | **Q** | **Years** | **Latitudinal range** | **Longitudinal range** | **spp** | ***n*** |
| --- | --- | --- | --- | --- | --- | --- | --- | --- | --- |
| BBICFraOT4 | France | GOV | Bay of Biscay | 4 | 1997-2020 | 43.69 to 48 | -7.72 to  -1.26 | 205 | 1766 |
| CSFraOT4 | France | GOV | Celtic Sea | 4 | 1997-2020 | 48 to 51.83 | -10.99 to  -5.77 | 116 | 1213 |
| CSIreOT4 | Ireland | GOV | Celtic Sea | 4 | 2003-2020 | 50.06 to 56.4 | -11.84 to  -5.09 | 154 | 2353 |
| CSNIrOT1 | N. Ireland | GOV | Celtic Sea | 1 | 2008-2020 | 52.29 to 54.73 | -6.11 to  -3.55 | 87 | 756 |
| CSNIrOT4 | N. Ireland | GOV | Celtic Sea | 4 | 2009-2020 | 52.29 to 54.73 | -6.11 to  -3.56 | 79 | 698 |
| CSScoOT1 | Scotland | GOV | Celtic Sea | 1 | 2011-2020 | 55 to 59.52 | -9.78 to  -4.03 | 101 | 484 |
| CSScoOT1  hist | Scotland | GOV | Celtic Sea | 1 | 1985-2010 | 55.11 to 59.64 | -9.39 to  -4.04 | 107 | 1054 |
| CSScoOT4 | Scotland | GOV | Celtic Sea | 4 | 2011-2020 | 54.26 to 59.99 | -10.63 to  -4.01 | 97 | 506 |
| CSScoOT4  hist | Scotland | GOV | Celtic Sea | 4 | 1997-2009 | 54.28 to 59.9 | -10.32 to  -4.03 | 101 | 704 |
| GNSFraOT4 | France | GOV | Greater North Sea | 4 | 1998-2020 | 49.32 to 50.99 | -1.64 to 1.66 | 107 | 1907 |
| GNSIntOT1 | International | GOV | Greater North Sea | 1 | 1983-2020 | 51.08 to 61.43 | -3.34 to 12.63 | 208 | 14380 |
| GNSIntOT1  channel | International | GOV | Greater North Sea | 1 | 2007-2020 | 49.6 to 50.98 | 0.02 to 1.66 | 79 | 192 |
| GNSIntOT3 | International | GOV | Greater North Sea | 3 | 1998-2020 | 51.53 to 61.37 | -3.36 to 12.63 | 170 | 7317 |
| WAScoOT3 | Scotland | GOV | Western Approaches | 3 | 2011-2020 | 56.12 to 57.91 | -15.23 to  -13.09 | 54 | 337 |
| WAScoOT3  hist | Scotland | GOV | Western Approaches | 3 | 1999-2009 | 56.2 to 57.91 | -15.23 to  -13.33 | 55 | 364 |
| BBICFraBT4 | France | Beam | Bay of Biscay | 4 | 2011-2020 | 44.39 to 47.88 | -4.61 to  -1.26 | 9 | 551 |
| CSEngBT1 | England | Beam | Celtic Sea | 1 | 2016-2019 | 48.56 to 51.94 | -8.71 to  -2.07 | 10 | 376 |
| CSEngBT3 | England | Beam | Celtic Sea | 3 | 1993-2019 | 52.1 to 54.8 | -6.19 to  -3.14 | 11 | 1653 |
| CSEngBT3  Bchannel | England | Beam | Celtic Sea | 3 | 1993-2020 | 50.53 to 51.69 | -5.54 to  -3.83 | 11 | 1008 |
| GNSBelBT3 | Belgium | Beam | Greater North Sea | 3 | 2004-2020 | 51.5 to 54 | 0.48 to 2.89 | 7 | 637 |
| GNSEngBT3 | England | Beam | Greater North Sea | 3 | 1990-2020 | 49.5 to 51 | -1.96 to 1.57 | 12 | 1692 |
| GNSGerBT3 | Germany | Beam | Greater North Sea | 3 | 1997-2020 | 55 to 57.28 | 4.08 to 8 | 11 | 1087 |
| GNSNetBT3 | Netherlands | Beam | Greater North Sea | 3 | 1999-2020 | 51.53 to 58.4 | -3.32 to 8.21 | 9 | 3068 |

Table S2. Taxonomic and size composition of feeding guilds (PL = planktivore, BE = benthivore, PI = piscivore). Values for species within guilds were calculated by taking the mean across unique predator samples. Prey weight = mean individual prey wet weight in g; PPMR = average biomass-weighted predator-prey mass ratios; ben = benthos prey; zoo = zooplankton prey. Sample sizes for prey weight and PPMR are provided in PPMR *n* and the relative contribution of prey functional groups in Diet *n*. ‘NA’ is used where data were not available, typically where prey mass information was missing and not possible to estimate.

| **Taxa** | **Guild** | **Length cm** | **Prey**  **weight**  **g** | **PPMR** |  | **PPMR *n*** | **% fish prey** | **% ben prey** | **% zoo prey** | **Diet *n*** |
| --- | --- | --- | --- | --- | --- | --- | --- | --- | --- | --- |
| *Ammodytes* | PL | 16.42 | 0.70 | 6426.72 |  | 282 | 50.21 | 12.31 | 25.17 | 1128 |
| *Arnoglossus laterna* | PL | 1.10 | >0.01 | 165.59 |  | 8 | 0.00 | 6.22 | 52.03 | 95 |
| *Belone belone* | PL | 24.31 | 1.25 | 682.93 |  | 6 | 28.81 | 30.32 | 12.55 | 1417 |
| *Callionymus lyra* | PL | 0.49 | >0.01 | 176.44 |  | 10 | 0.00 | 7.01 | 66.22 | 224 |
| *Clupea harengus* | PL | 22.83 | 0.09 | 457816.45 |  | 1460 | 7.61 | 5.96 | 43.48 | 16252 |
| *Engraulis encrasicolus* | PL | 18.13 | 0.72 | 76.23 |  | 67 | 80.62 | 1.20 | 2.81 | 39 |
| *Gadiculus argenteus* | PL | 1.50 | NA | NA |  | NA | NA | NA | NA | 91 |
| *Gadus morhua* | PL | 2.20 | >0.01 | 554.55 |  | 170 | 0.00 | 0.75 | 72.55 | 320 |
| *Glyptocephalus cynoglossus* | PL | 1.70 | >0.01 | 339.91 |  | 7 | 0.00 | 0.00 | 79.48 | 48 |
| Gobiidae | PL | 2.49 | 0.05 | 1410.55 |  | 57 | 0.00 | 21.04 | 38.69 | 4809 |
| *Limanda limanda* | PL | 3.22 | 0.15 | 280.67 |  | 22 | 0.33 | 36.20 | 13.05 | 2108 |
| *Melanogrammus aeglefinus* | PL | 2.40 | >0.01 | 591.56 |  | 45 | 0.00 | 7.85 | 54.65 | 83 |
| *Merlangius merlangus* | PL | 7.24 | 0.07 | 1970.08 |  | 2791 | 7.61 | 14.35 | 44.72 | 14861 |
| *Micromesistius poutassou* | PL | 21.66 | 0.27 | 32603.58 |  | 647 | 7.70 | 0.65 | 61.46 | 703 |
| *Microstomus kitt* | PL | 0.94 | >0.01 | 169.26 |  | 4 | 0.00 | 18.56 | 79.84 | 139 |
| *Molva molva* | PL | 1.24 | >0.01 | 82.20 |  | 5 | 0.00 | 0.00 | 68.43 | 48 |
| *Myctophidae* | PL | 1.50 | >0.01 | 328.60 |  | 1 | 0.00 | 0.00 | 64.85 | 36 |
| *Pholis gunnellus* | PL | 1.72 | >0.01 | 1061.02 |  | 8 | 0.00 | 8.29 | 60.02 | 44 |
| *Pollachius pollachius* | PL | 11.34 | 0.22 | 133.16 |  | 10 | 5.90 | 41.02 | 27.42 | 469 |
| *Pollachius virens* | PL | 7.37 | 0.32 | 1753.20 |  | 121 | 11.59 | 5.38 | 56.36 | 11865 |
| *Sardina pilchardus* | PL | 1.79 | >0.01 | 2380.49 |  | 2 | 0.00 | 0.00 | 46.51 | 40 |
| *Scomber scombrus* | PL | 33.14 | 0.67 | 297117.67 |  | 8233 | 14.00 | 3.02 | 38.01 | 16555 |
| *Sprattus sprattus* | PL | 9.33 | 0.16 | 3230.27 |  | 458 | 26.66 | 6.54 | 37.26 | 1812 |
| *Trachurus trachurus* | PL | 13.70 | 0.07 | 18872.63 |  | 45 | 5.26 | 13.19 | 30.24 | 297 |
| *Trisopterus esmarkii* | PL | 12.79 | 0.07 | 3734.61 |  | 289 | 2.34 | 3.54 | 63.42 | 5592 |
| *Agonus cataphractus* | BE | 12.12 | 0.03 | 1048.33 |  | 26 | 0.97 | 60.79 | 0.00 | 194 |
| *Anguilla anguilla* | BE | 40.90 | 2.47 | 10765.26 |  | 14 | 8.84 | 84.57 | 0.00 | 209 |
| *Argentina sphyraena* | BE | 19.89 | 1.92 | 837.86 |  | 21 | 0.98 | 23.25 | 18.75 | 509 |
| *Callionymus lyra* | BE | 18.36 | 0.18 | 2213.06 |  | 70 | 0.00 | 39.68 | 2.05 | 430 |
| *Ciliata mustela* | BE | 14.97 | 1.18 | 148.15 |  | 36 | 2.95 | 37.50 | 0.00 | 235 |
| *Coryphoblennius galerita* | BE | 5.12 | NA | NA |  | NA | NA | NA | NA | 1174 |
| *Ctenolabrus rupestris* | BE | 3.67 | NA | NA |  | NA | NA | NA | NA | 35 |
| *Dicentrarchus labrax* | BE | 15.59 | 2.53 | 1349.16 |  | 28 | 10.52 | 62.97 | 1.89 | 5444 |
| *Enchelyopus cimbrius* | BE | 20.15 | 0.34 | 1829.87 |  | 28 | 2.72 | 28.04 | 6.78 | 240 |
| *Glyptocephalus cynoglossus* | BE | 35.16 | 1.30 | 1435.74 |  | 7 | 3.98 | 32.33 | 2.07 | 63 |
| Gobiidae | BE | 7.23 | 0.14 | 947.67 |  | 17 | 0.46 | 55.82 | 0.53 | 1130 |
| *Hippoglossoides platessoides* | BE | 14.11 | 1.12 | 146.83 |  | 15 | 15.31 | 21.26 | 0.01 | 254 |
| *Lepidion eques* | BE | 21.30 | NA | NA |  | NA | NA | NA | NA | 2562 |
| *Limanda limanda* | BE | 20.78 | 1.15 | 2123.71 |  | 1897 | 5.87 | 67.55 | 0.32 | 11014 |
| *Lipophrys pholis* | BE | 4.36 | 0.08 | 135.95 |  | 52 | 0.00 | 85.78 | 1.50 | 4622 |
| *Melanogrammus aeglefinus* | BE | 31.67 | 0.91 | 5161.70 |  | 10092 | 13.60 | 53.15 | 6.56 | 64679 |
| *Microstomus kitt* | BE | 27.24 | 1.38 | 10696.37 |  | 105 | 0.64 | 47.22 | 0.42 | 1071 |
| *Myoxocephalus scorpius* | BE | 18.89 | 3.70 | 73.02 |  | 12 | 25.06 | 55.12 | 0.00 | 278 |
| *Pholis gunnellus* | BE | 12.31 | 0.17 | 706.18 |  | 43 | 0.17 | 60.05 | 0.01 | 1234 |
| *Platichthys flesus* | BE | 18.62 | 0.64 | 3813.51 |  | 226 | 10.85 | 62.17 | 2.12 | 4201 |
| *Pleuronectes platessa* | BE | 28.14 | 0.51 | 2826.79 |  | 1434 | 6.20 | 61.04 | 1.09 | 29928 |
| *Salmo trutta* | BE | 38.12 | 1.18 | 80907.01 |  | 22 | 82.36 | 2.81 | 1.36 | 249 |
| *Scophthalmus rhombus* | BE | 10.95 | 0.36 | 176.23 |  | 36 | 62.58 | 19.45 | 0.00 | 203 |
| *Scyliorhinus stellaris* | BE | 77.03 | NA | NA |  | NA | NA | NA | NA | 110 |
| *Solea solea* | BE | 28.91 | 0.66 | 4139.47 |  | 80 | 17.51 | 37.68 | 0.49 | 2132 |
| *Taurulus bubalis* | BE | 5.20 | 0.24 | 202.85 |  | 31 | 4.22 | 64.22 | 1.99 | 1549 |
| *Trachinus draco* | BE | 26.20 | 4.71 | 26.70 |  | 2 | 100.00 | 0.00 | 0.00 | 82 |
| *Trisopterus luscus* | BE | 8.39 | 0.22 | 232.89 |  | 16 | 0.00 | 31.05 | 16.43 | 405 |
| *Amblyraja radiata* | PI | 35.96 | 1.45 | 6014.07 |  | 716 | 31.05 | 30.34 | 1.91 | 3551 |
| *Arnoglossus laterna* | PI | 13.07 | 0.30 | 854.27 |  | 25 | 10.10 | 43.81 | 3.09 | 290 |
| *Buglossidium luteum* | PI | 9.24 | 0.02 | 631.39 |  | 1 | 0.00 | 100.00 | 0.00 | 268 |
| *Chelidonichthys cuculus* | PI | 23.56 | 1.43 | 1201.79 |  | 269 | 11.98 | 29.50 | 8.56 | 1193 |
| *Chelidonichthys lucerna* | PI | NA | NA | NA |  | NA | NA | NA | NA | 804 |
| *Dipturus batis* | PI | 70.74 | NA | NA |  | NA | NA | NA | NA | 96 |
| *Echiichthys vipera* | PI | 12.11 | 0.64 | 516.36 |  | 336 | 42.38 | 4.15 | 0.99 | 813 |
| *Eutrigla gurnardus* | PI | 22.88 | 1.25 | 1843.18 |  | 3283 | 39.11 | 13.11 | 5.51 | 22587 |
| *Gadus morhua* | PI | 53.32 | 11.02 | 4270.73 |  | 22722 | 41.73 | 25.45 | 15.25 | 96627 |
| *Galeorhinus galeus* | PI | 75.18 | NA | NA |  | NA | NA | NA | NA | 90 |
| *Hippoglossoides platessoides* | PI | 20.96 | 0.86 | 645.07 |  | 306 | 40.60 | 30.29 | 2.88 | 1949 |
| *Lepidorhombus boscii* | PI | 20.66 | NA | NA |  | NA | NA | NA | NA | 57 |
| *Lepidorhombus whiffiagonis* | PI | 32.28 | 7.01 | 509.95 |  | 1868 | 58.74 | 11.80 | 4.64 | 1882 |
| *Leucoraja naevus* | PI | 41.03 | 2.11 | 1572.50 |  | 119 | 24.83 | 14.13 | 3.15 | 363 |
| *Lophius budegassa* | PI | 25.49 | NA | NA |  | NA | NA | NA | NA | 30 |
| *Lophius piscatorius* | PI | 40.91 | 39.59 | 116.20 |  | 538 | 89.84 | 2.27 | 0.01 | 1131 |
| *Merlangius merlangus* | PI | 27.18 | 2.64 | 3070.90 |  | 13629 | 50.08 | 11.49 | 6.40 | 145609 |
| *Merluccius merluccius* | PI | 42.50 | 15.46 | 125.07 |  | 1645 | 82.83 | 0.71 | 5.23 | 11424 |
| *Microchirus variegatus* | PI | 14.08 | 0.02 | 6968.54 |  | 5 | 0.00 | 24.33 | 1.72 | 812 |
| *Molva molva* | PI | 56.85 | 15.72 | 1070.30 |  | 27 | 83.29 | 4.90 | 0.00 | 45 |
| *Mullus surmuletus* | PI | 26.47 | 0.57 | 414.81 |  | 2 | 0.00 | 79.05 | 0.00 | 33 |
| *Pollachius pollachius* | PI | 56.64 | 17.68 | 27032.92 |  | 72 | 51.32 | 16.58 | 9.86 | 871 |
| *Pollachius virens* | PI | 62.24 | 14.03 | 5986.60 |  | 1459 | 61.17 | 0.81 | 22.01 | 13447 |
| *Prionace glauca* | PI | 169.68 | NA | NA |  | NA | NA | NA | NA | 451 |
| *Psetta maxima* | PI | 33.69 | 9.33 | 619.26 |  | 377 | 84.41 | 4.98 | 0.41 | 4813 |
| *Raja brachyura* | PI | 32.11 | 6.07 | 215.09 |  | 1 | 100.00 | 0.00 | 0.00 | 116 |
| *Raja clavata* | PI | 47.12 | 3.12 | 1405.16 |  | 295 | 11.65 | 54.31 | 2.38 | 2902 |
| *Raja montagui* | PI | 41.99 | 2.32 | 2387.31 |  | 104 | 14.74 | 39.22 | 0.79 | 423 |
| *Scophthalmus rhombus* | PI | 36.09 | 6.68 | 397.65 |  | 165 | 88.04 | 1.41 | 0.00 | 1451 |
| *Scyliorhinus canicula* | PI | 52.14 | 9.25 | 1458.11 |  | 319 | 36.65 | 38.25 | 2.01 | 2041 |
| *Squalus acanthias* | PI | 67.07 | 47.82 | 950.39 |  | 305 | 67.58 | 5.53 | 0.20 | 1612 |
| *Trachurus trachurus* | PI | 29.71 | 0.27 | 108152.58 |  | 492 | 14.32 | 10.63 | 27.45 | 4838 |
| *Trisopterus luscus* | PI | 24.03 | 1.36 | 2076.80 |  | 207 | 14.99 | 46.24 | 0.88 | 1681 |
| *Trisopterus minutus* | PI | 15.35 | 0.54 | 956.26 |  | 338 | 7.36 | 19.22 | 12.04 | 1681 |
| *Zeus faber* | PI | 32.99 | 6.68 | 166.53 |  | 86 | 92.61 | 0.48 | 0.00 | 265 |


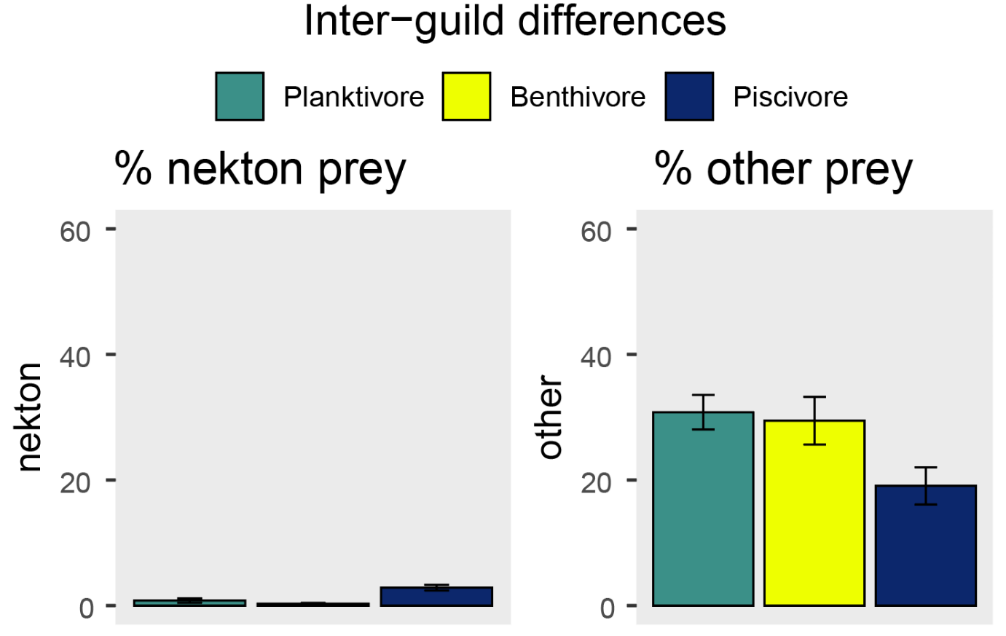


Fig. S1. Remaining differences between guilds in the % biomass contribution of different prey functional groups (see Fig. 1 for main prey functional groups). Prey classified as ‘other’ consisted of those which could not be classified into World Register of Marine Species functional groups (99.7%) with some remaining that were overly rare to be included as individual groups (e.g. birds, meiofauna; 0.3%). Values are based on means taken across species within guilds, error bars represent standard error.


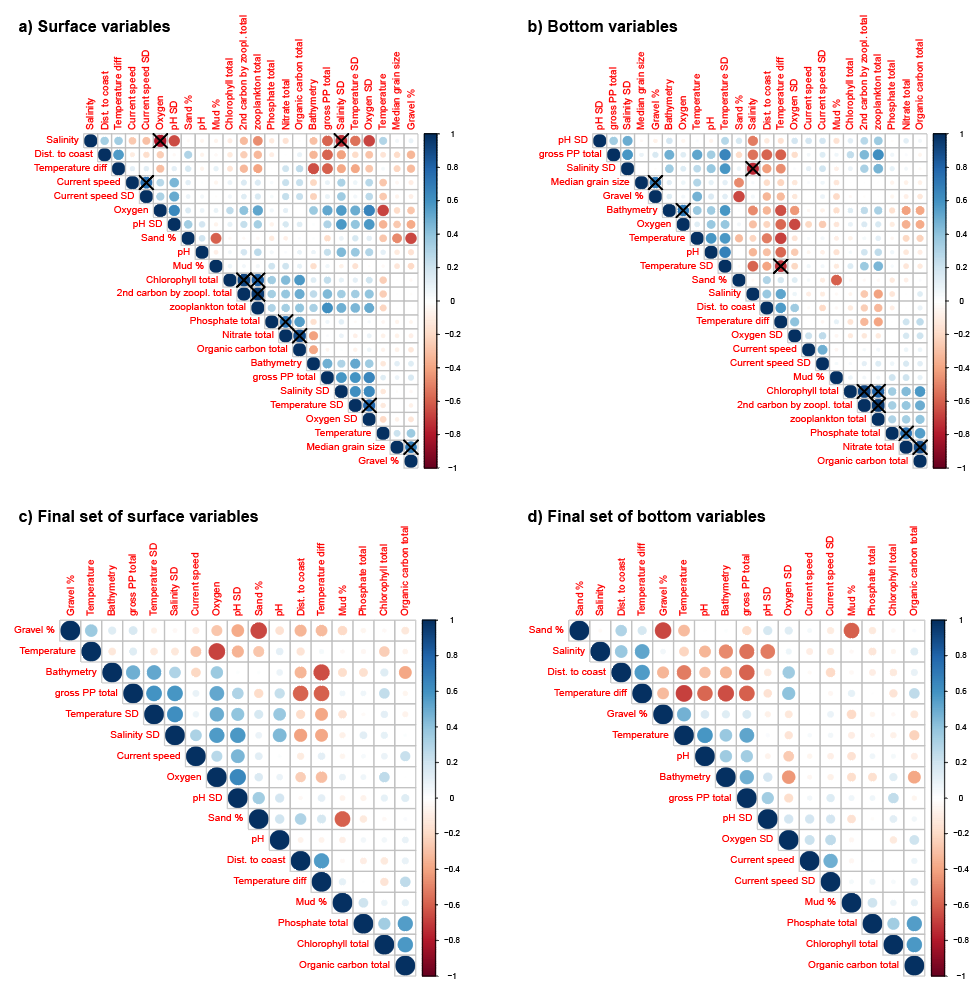


Fig. S2. Pairwise Pearson correlation coefficients for all covariates considered were evaluated based on where we had trawl observations. Black crosses highlight correlations >0.7 using the full set of environmental variables considered (a, b) where we chose to exclude one of the covarying variables (c, d). We use sea surface values (a, c) to model habitat suitability for planktivores, which are largely pelagic species, and seabed values (b, d) for the benthivores and piscivores which are largely demersal species. “Gross PP” = gross primary production; “2^nd^ carbon by zoopl." = secondary production by zooplankton; “Organic carbon” = non-living organic carbon; “Temperature diff” = the difference between surface and bottom temperature (i.e., a measure of stratification; The suffix “SD” represents a measure of spatio-temporal heterogeneity, i.e., the standard deviation of the 12 monthly means in each year, for all locations within a radius of 75 km of each grid cell; The suffix “total” denotes the variable does not refer to the surface or bottom but has instead been summed up across the water column.


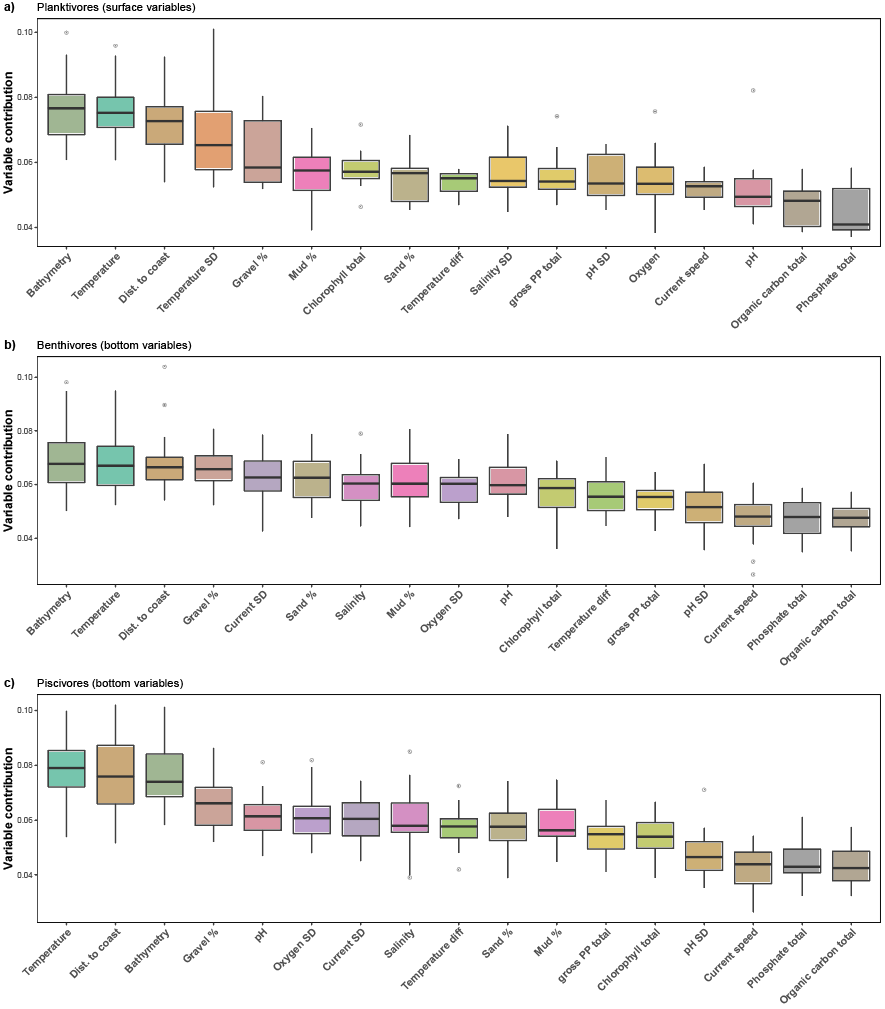


Fig. S3. Variable importance as measured in the proportion of total branches used for a given variable across our BART models, split by feeding guild. This shows which variables best explain the spatial distributions of species within feeding guilds.


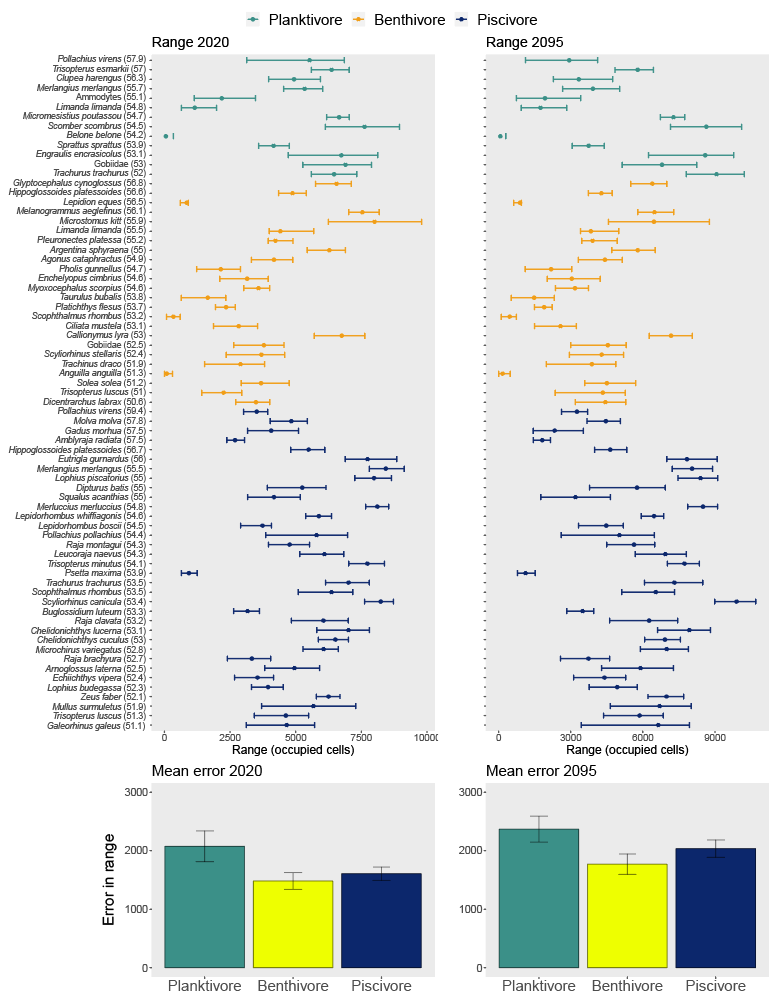


Fig. S4. Top plots: prediction errors for the range occupied by species within guilds with error bars representing the minimum and maximum estimated range in cells occupied, based on the 0.15865 quantile (lower error) and 0.84135 quantile (upper error) of the uncertainty in the BART model projections. Bottom plots: guild-level mean error with error bars representing standard error. Uncertainty is higher, on average, for planktivores, relative to benthivore and piscivore predictions.


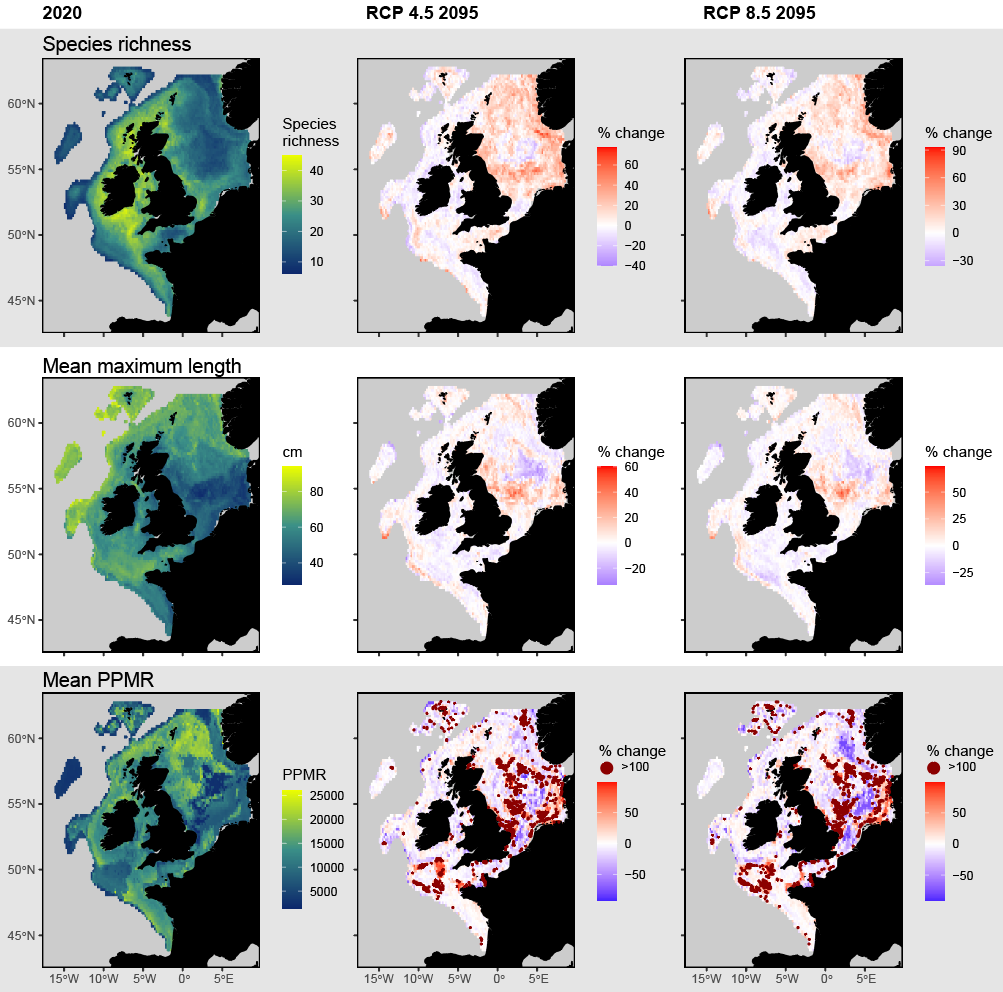


Fig. S5. Across-guild species richness, mean maximum length and predator-prey mass ratios (PPMR) in 2020 (left column), and % change between 2020 and 2095 based on RCP4.5 (middle column) and RCP8.5 (right column) generated using BART species distribution models. Mean maximum length and PPMR values are based on means taken across species within guilds in each grid cell (i.e., PPMR values reported in Table S2). Particularly high values of increase by >100% are highlighted in dark red.

Table S3. Model performance, size classes and *n* of ‘presence’ observations in the trawl survey data used for model training, for all combinations of taxa and guilds (PL = planktivore, BE = benthivore, PI = piscivore). Model performance was assessed as the Area Under the Curve (AUC) of the Receiver-Operating Characteristic (ROC) and Precision-Recall (PR) curves and the models’ Miller slope, all of them measured on evaluation data for both “novel space” and “novel time” (see main manuscript for details of how this was done). A total of 9*71= 639 additional models were trained for model validation (8 for spatial block cross validation, 1 for temporal validation). Only models with an AUC ROC of 0.65 or above in both the spatial and the temporal evaluation were used in our Results. Those species and sizes with AUC ROC values <0.65 or with insufficient *n* observations for modelling are highlighted in bold. Size classes are determined as follows: <3 cm as larvae (Lv); small juvenile fish between 3 cm and half of length at maturity (Js); juvenile-medium fish from half of length at maturity to length at maturity (Jm); medium fish from length at maturity to half-maximum length (M); and all remaining larger fish as large (L).

|  |  |  |  | SPACE | | | | TIME | |
| --- | --- | --- | --- | --- | --- | --- | --- | --- | --- |
| Taxa | Guild | Size classes | n | AUC ROC | AUC PR | Miller slope | AUC ROC | AUC PR | Miller slope |
| Ammodytes | PL | Lv, Jm, M, L | 4047 | 0.74 | 0.34 | 0.74 | 0.80 | 0.43 | 0.86 |
| *Belone belone* | PL | Lv, Js, L | 44 | 0.73 | 0.01 | 0.77 | 0.78 | 0.00 | 0.79 |
| *Clupea harengus* | PL | Lv, Js, Jm, M, L | 103185 | 0.83 | 0.88 | 0.82 | 0.88 | 0.89 | 0.91 |
| *Engraulis encrasicolus* | PL | L | 1475 | 0.71 | 0.11 | 0.62 | 0.74 | 0.31 | 0.83 |
| Gobiidae | PL | Lv, Js, Jm | 8880 | 0.75 | 0.20 | 0.62 | 0.73 | 0.34 | 0.67 |
| *Limanda limanda* | PL | Lv, Js | 5548 | 0.67 | 0.46 | 0.50 | 0.81 | 0.50 | 0.79 |
| *Merlangius merlangus* | PL | Lv, Js | 22718 | 0.78 | 0.65 | 0.79 | 0.83 | 0.73 | 0.80 |
| *Micromesistius poutassou* | PL | Jm, M, L | 22260 | 0.95 | 0.78 | 0.97 | 0.94 | 0.83 | 0.78 |
| *Pollachius virens* | PL | Lv, Js | 438 | 0.80 | 0.09 | 0.95 | 0.84 | 0.12 | 0.88 |
| *Scomber scombrus* | PL | Jm, M, L | 37791 | 0.70 | 0.61 | 0.68 | 0.68 | 0.69 | 0.65 |
| *Sprattus sprattus* | PL | Lv, Js, Jm, M, L | 63050 | 0.85 | 0.76 | 0.78 | 0.91 | 0.86 | 0.92 |
| *Trachurus trachurus* | PL | Js, Jm | 19632 | 0.80 | 0.67 | 0.84 | 0.83 | 0.81 | 0.98 |
| *Trisopterus esmarkii* | PL | Lv, Js, Jm, M, L | 71165 | 0.93 | 0.87 | 0.94 | 0.93 | 0.92 | 0.90 |
| *Agonus cataphractus* | BE | M, L | 5786 | 0.78 | 0.23 | 0.56 | 0.85 | 0.41 | 1.00 |
| *Anguilla anguilla* | BE | Jm, M | 64 | 0.76 | 0.01 | 0.62 | 0.80 | 0.04 | 1.11 |
| *Argentina sphyraena* | BE | M, L | 16636 | 0.90 | 0.74 | 0.85 | 0.92 | 0.81 | 0.99 |
| *Callionymus lyra* | BE | Jm, M, L | 37761 | 0.77 | 0.68 | 0.83 | 0.81 | 0.79 | 0.83 |
| *Ciliata mustela* | BE | Js, Jm | 808 | 0.84 | 0.04 | 0.49 | 0.92 | 0.19 | 1.04 |
| *Dicentrarchus labrax* | BE | Lv, Js, Jm, M, L | 4788 | 0.93 | 0.34 | 0.89 | 0.96 | 0.51 | 1.07 |
| *Enchelyopus cimbrius* | BE | Js, Jm | 6026 | 0.82 | 0.38 | 0.68 | 0.88 | 0.42 | 0.91 |
| *Glyptocephalus cynoglossus* | BE | M, L | 9694 | 0.84 | 0.49 | 0.77 | 0.88 | 0.65 | 0.94 |
| Gobiidae | BE | M, L | 2582 | 0.85 | 0.16 | 0.67 | 0.92 | 0.45 | 0.93 |
| *Hippoglossoides platessoides* | BE | Js, M, L | 42498 | 0.90 | 0.86 | 0.86 | 0.95 | 0.91 | 1.05 |
| *Lepidion eques* | BE | Jm, M | 86 | 0.99 | 0.11 | 1.10 | 1.00 | 0.66 | 1.70 |
| *Limanda limanda* | BE | Jm, M, L | 22677 | 0.90 | 0.65 | 0.86 | 0.97 | 0.99 | 1.30 |
| *Melanogrammus aeglefinus* | BE | Js, Jm, M, L | 78144 | 0.95 | 0.70 | 0.90 | 0.97 | 0.98 | 0.99 |
| *Microstomus kitt* | BE | Jm, M, L | 13425 | 0.81 | 0.76 | 0.70 | 0.89 | 0.87 | 1.08 |
| *Myoxocephalus scorpius* | BE | M, L | 3786 | 0.86 | 0.25 | 0.61 | 0.92 | 0.34 | 0.84 |
| *Pholis gunnellus* | BE | Jm, M | 424 | 0.87 | 0.04 | 0.73 | 0.92 | 0.26 | 1.26 |
| *Platichthys flesus* | BE | Lv, Js, Jm, M, L | 13825 | 0.92 | 0.51 | 0.93 | 0.95 | 0.53 | 1.09 |
| *Pleuronectes platessa* | BE | Lv, Js, Jm, M, L | 32512 | 0.92 | 0.49 | 0.82 | 0.96 | 0.99 | 0.95 |
| *Scophthalmus rhombus* | BE | Js, Jm | 56 | 0.92 | 0.01 | 1.13 | 0.68 | 0.01 | 0.96 |
| *Scyliorhinus stellaris* | BE | M, L | 1342 | 0.90 | 0.27 | 0.76 | 0.95 | 0.50 | 0.97 |
| *Solea solea* | BE | Js, Jm, M, L | 27664 | 0.84 | 0.86 | 0.77 | 0.91 | 0.96 | 1.02 |
| *Taurulus bubalis* | BE | Js, Jm, M | 567 | 0.80 | 0.02 | 0.44 | 0.93 | 0.13 | 1.15 |
| *Trachinus draco* | BE | M, L | 964 | 0.88 | 0.21 | 0.74 | 0.96 | 0.53 | 1.24 |
| *Trisopterus luscus* | BE | Js | 492 | 0.92 | 0.19 | 0.76 | 0.97 | 0.32 | 1.18 |
| *Amblyraja radiata* | PI | Js, Jm, M | 17829 | 0.89 | 0.57 | 1.00 | 0.92 | 0.50 | 0.92 |
| *Arnoglossus laterna* | PI | M, L | 7714 | 0.82 | 0.36 | 0.79 | 0.86 | 0.49 | 0.88 |
| *Buglossidium luteum* | PI | M | 3197 | 0.90 | 0.48 | 0.82 | 0.93 | 0.62 | 0.80 |
| *Chelidonichthys cuculus* | PI | Js, Jm, M, L | 29952 | 0.88 | 0.69 | 0.65 | 0.93 | 0.85 | 0.95 |
| *Chelidonichthys lucerna* | PI | Jm, M, L | 8517 | 0.79 | 0.21 | 0.69 | 0.84 | 0.46 | 0.98 |
| *Dipturus batis* | PI | Js, Jm | 1394 | 0.80 | 0.07 | 0.61 | 0.88 | 0.14 | 1.00 |
| *Echiichthys vipera* | PI | Js, Jm | 5064 | 0.84 | 0.25 | 0.58 | 0.91 | 0.53 | 0.81 |
| *Eutrigla gurnardus* | PI | Js, Jm, M, L | 83012 | 0.76 | 0.83 | 0.70 | 0.84 | 0.93 | 0.88 |
| *Gadus morhua* | PI | Js, Jm, M, L | 70608 | 0.75 | 0.79 | 0.69 | 0.82 | 0.80 | 0.98 |
| *Galeorhinus galeus* | PI | Js, Jm | 704 | 0.79 | 0.08 | 0.74 | 0.87 | 0.19 | 1.07 |
| *Hippoglossoides platessoides* | PI | Jm | 15986 | 0.91 | 0.88 | 0.87 | 0.95 | 0.92 | 1.07 |
| *Lepidorhombus boscii* | PI | M | 1318 | 0.97 | 0.69 | 0.82 | 0.99 | 0.92 | 1.28 |
| *Lepidorhombus whiffiagonis* | PI | Jm, M, L | 26073 | 0.94 | 0.78 | 0.96 | 0.98 | 0.96 | 1.04 |
| *Leucoraja naevus* | PI | Js, Jm | 9368 | 0.81 | 0.37 | 0.70 | 0.88 | 0.55 | 1.01 |
| *Lophius budegassa* | PI | Jm | 1316 | 0.93 | 0.33 | 0.91 | 0.94 | 0.49 | 0.93 |
| *Lophius piscatorius* | PI | Js, Jm, M | 28428 | 0.83 | 0.63 | 0.96 | 0.81 | 0.69 | 0.76 |
| *Merlangius merlangus* | PI | Jm, M, L | 75303 | 0.84 | 0.68 | 0.70 | 0.95 | 0.98 | 1.00 |
| *Merluccius merluccius* | PI | Js, Jm, M, L | 44724 | 0.92 | 0.76 | 0.86 | 0.95 | 0.94 | 1.00 |
| *Microchirus variegatus* | PI | Jm, M | 9366 | 0.91 | 0.59 | 0.87 | 0.91 | 0.65 | 0.92 |
| *Molva molva* | PI | Jm | 2971 | 0.84 | 0.34 | 0.87 | 0.90 | 0.50 | 1.04 |
| *Mullus surmuletus* | PI | L | 1097 | 0.74 | 0.07 | 0.62 | 0.86 | 0.27 | 1.20 |
| *Pollachius pollachius* | PI | Jm, M, L | 2085 | 0.74 | 0.07 | 0.66 | 0.82 | 0.11 | 0.99 |
| *Pollachius virens* | PI | Jm, M, L | 14328 | 0.91 | 0.67 | 1.09 | 0.94 | 0.79 | 1.01 |
| *Psetta maxima* | PI | Js, Jm, M, L | 7868 | 0.71 | 0.43 | 0.61 | 0.82 | 0.61 | 1.02 |
| *Raja brachyura* | PI | Js, Jm | 1426 | 0.87 | 0.14 | 0.78 | 0.90 | 0.32 | 1.03 |
| *Raja clavata* | PI | Js, Jm, M | 11712 | 0.80 | 0.33 | 0.65 | 0.88 | 0.65 | 1.09 |
| *Raja montagui* | PI | Js, Jm, M | 10566 | 0.85 | 0.47 | 0.71 | 0.90 | 0.72 | 1.02 |
| *Scophthalmus rhombus* | PI | M, L | 2382 | 0.76 | 0.09 | 0.66 | 0.84 | 0.30 | 1.11 |
| *Scyliorhinus canicula* | PI | Jm, M, L | 36582 | 0.89 | 0.81 | 0.86 | 0.93 | 0.94 | 1.01 |
| *Squalus acanthias* | PI | Js, Jm, M, L | 15616 | 0.75 | 0.30 | 0.72 | 0.84 | 0.50 | 1.08 |
| *Trachurus trachurus* | PI | M, L | 22268 | 0.71 | 0.58 | 0.65 | 0.80 | 0.75 | 1.03 |
| *Trisopterus luscus* | PI | Jm, M, L | 8193 | 0.83 | 0.30 | 0.60 | 0.93 | 0.54 | 1.26 |
| *Trisopterus minutus* | PI | Jm, M, L | 45069 | 0.83 | 0.81 | 0.87 | 0.88 | 0.86 | 0.89 |
| *Zeus faber* | PI | Js, Jm, M, L | 21560 | 0.85 | 0.55 | 0.75 | 0.89 | 0.65 | 1.00 |
| ***Ctenolabrus rupestris*** | **BE** | **Js** | **0** | **NA** | **NA** | **NA** | **NA** | **NA** | **NA** |
| ***Gadiculus argenteus*** | **PL** | **Lv** | **0** | **NA** | **NA** | **NA** | **NA** | **NA** | **NA** |
| ***Myctophidae*** | **PL** | **Lv** | **0** | **NA** | **NA** | **NA** | **NA** | **NA** | **NA** |
| ***Gadus morhua*** | **PL** | **Lv** | **1** | **NA** | **NA** | **NA** | **NA** | **NA** | **NA** |
| ***Pholis gunnellus*** | **PL** | **Lv** | **1** | **NA** | **NA** | **NA** | **NA** | **NA** | **NA** |
| ***Arnoglossus laterna*** | **PL** | **Lv** | **2** | **NA** | **NA** | **NA** | **NA** | **NA** | **NA** |
| ***Sardina pilchardus*** | **PL** | **Lv** | **2** | **NA** | **NA** | **NA** | **NA** | **NA** | **NA** |
| ***Callionymus lyra*** | **PL** | **Lv** | **3** | **NA** | **NA** | **NA** | **NA** | **NA** | **NA** |
| ***Pollachius pollachius*** | **PL** | **Lv, Js** | **10** | **NA** | **NA** | **NA** | **NA** | **NA** | **NA** |


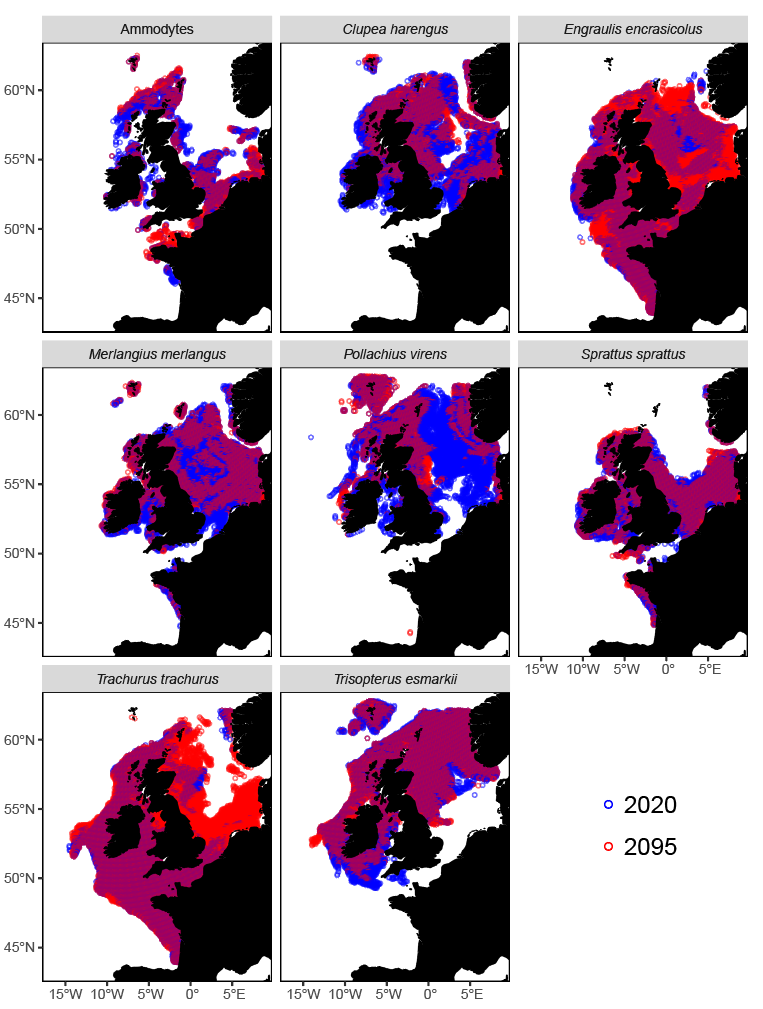


Fig. S6. Change in the distribution of some key planktivores, including juvenile life stage of species that develop into piscivores as they mature (*Trachurus trachurus, Merlangius merlangus and Pollachius virens*), between 2020 (blue) and 2095 (red) under RCP 8.5.


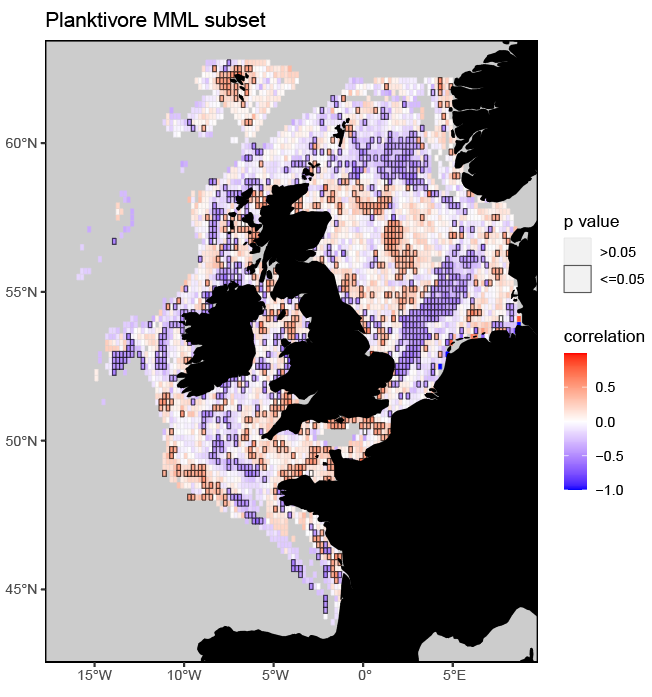


Fig. S7. Temporal correlations in the mean maximum length of planktivores that remain planktivorous through ontogeny over five-year intervals from 2020 to 2095 under RCP 8.5. Temporal increases are shown by red cells (Kendall’s tau correlation values between 0 and +1), declines by blue cells (correlation values between 0 and -1), and cells with significant correlations have a black border.

References

Lynam, C. P. & Ribeiro, J. *A data product derived from Northeast Atlantic groundfish data from scientific trawl surveys 1983-2020*. (2022). doi:https://doi.org/10.14466/CefasDataHub.126.
